# Efficacy and safety of intravitreal aflibercept in ranibizumab-refractory patients with neovascular age-related macular degeneration

**DOI:** 10.1186/s12886-021-01841-6

**Published:** 2021-02-17

**Authors:** Sam Razavi, Laurent Kodjikian, Audrey Giocanti-Aurégan, Ingrid Dufour, Eric Souied

**Affiliations:** 1 Centre d’Ophtalmologie, 30 Boulevard Heurteloup, 37000 Tours, France; 2 Centre Hospitalier de la Cr Rousse, Université de Lyon, Lyon, France; 3 Hôpital Avicenne, AP-HP, Université Paris 13, DHU Vision et Handicaps, Bobigny, France; 4 Bayer HealthCare SAS, 220 Avenue de la Recherche, 59120 Loos, France; 5 Centre Hospitalier Intercommunal de Créteil, 40 Verdun avenue, Créteil, 94000 Paris, France

**Keywords:** Retina, Age-related macular degeneration, Exudative, Switch, Observational

## Abstract

**Background:**

Anti–vascular endothelial growth factor (anti-VEGF) agents have become the standard of care in neovascular age-related macular degeneration (nAMD). Despite generally excellent response rates to anti-VEGF therapy, some patients do not respond or may respond suboptimally. In the case of refractory or rapidly recurring fluid in nAMD, clinicians may switch to another anti-VEGF agent. TITAN was an observational study that assessed the effectiveness and safety of intravitreal aflibercept (IVT-AFL) in patients with nAMD refractory to ranibizumab who switched to IVT-AFL after less than 12 months of ranibizumab treatment in routine clinical practice in France.

**Methods:**

TITAN was an observational, retrospective and prospective 12-month study conducted at 28 centres in France. Patients with nAMD refractory to ranibizumab were enrolled. Patients who were switched from ranibizumab to IVT-AFL were followed for 12 months. Data were obtained from medical records for retrospectively included patients, and at routine follow-up visits for those included prospectively. The main outcome measure was percentage of patients who achieved treatment success (gain of ≥1 Early Treatment Diabetic Retinopathy Study letters in best-corrected visual acuity [BCVA] and/or any reduction in central retinal thickness [CRT]) from baseline to 12 months after switching. A sample size of 225 patients was determined based on a 2-sided 95% confidence interval with a width equal to 0.12 when the sample proportion was 0.70.

**Results:**

We analysed safety data (*N* = 217) and clinical outcomes from patients in the per-protocol population (*n* = 125). The mean (standard deviation) number of IVT-AFL injections was 7.5 (2.6). Treatment success was achieved in 68.8% of patients. Mean BCVA change from baseline to Month 12 was + 1.5 letters (*P* = 0.105) and the mean CRT change was − 45.0 μm (*P* <  0.001). In a subgroup analysis, in patients who received three initial monthly IVT-AFL injections, mean BCVA gain was 3.3 letters at Month 12 (*P* = 0.015). Excluding lack of efficacy and inappropriate scheduling of drug administration, the most common adverse event was eye pain (2.3%).

**Conclusions:**

Switching ranibizumab-refractory patients with nAMD to IVT-AFL may improve visual outcomes in some patients, particularly those who receive three initial monthly injections.

**Trial registration:**

ClinicalTrials.gov, NCT02321241. First posted: December 22, 2014; Last update posted: July 2, 2018

**Supplementary Information:**

The online version contains supplementary material available at 10.1186/s12886-021-01841-6.

## Background

Neovascular age-related macular degeneration (nAMD) is the most severe form of AMD and is the most common cause of legal blindness [1, 2]. Anti–vascular endothelial growth factor (anti-VEGF) agents, including intravitreal aflibercept (IVT-AFL) and ranibizumab, have become standard of care in nAMD [3–8]. Despite generally excellent response rates to anti-VEGF therapy, some patients do not respond or may respond suboptimally [3–8]. Some studies have shown reduced anatomic response over time with ranibizumab treatment in patients with nAMD, and there have been reports of loss of bioefficacy after repeated ranibizumab treatment [9–11].

In the case of refractory or rapidly recurring fluid in nAMD, clinicians may switch from the current anti-VEGF agent to another anti-VEGF agent [12]. The VIEW 1 and VIEW 2 studies [13] assessed the efficacy and safety of IVT-AFL in patients with nAMD and demonstrated non-inferiority of IVT-AFL 2 mg, given every 8 weeks after three initial monthly doses, versus ranibizumab 0.5 mg, every 4 weeks, in maintaining vision (loss of < 15 Early Treatment Diabetic Retinopathy [ETDRS] letters in best-corrected visual acuity [BCVA]) in treatment-naïve patients over a 12-month period [14–17].

Retrospective studies have shown that individualised IVT-AFL treatment can significantly reduce retinal fluid and preserve vision in patients with nAMD who are resistant to anti-VEGF agents [17–20]. However, prospective studies examining a switch to IVT-AFL in patients refractory to ranibizumab treatment in a real-world setting are scarce [21].

TITAN was an observational study that assessed the effectiveness and safety of IVT-AFL in patients with nAMD refractory to ranibizumab (persistence of intraretinal [IRF] and/or subretinal fluid [SRF]) who switched to IVT-AFL after less than 12 months of ranibizumab treatment in routine clinical practice in France.

## Methods

### Study design

TITAN (NCT02321241) was an observational 12-month study to assess the effectiveness and safety of IVT-AFL in patients with nAMD refractory to ranibizumab. The study was conducted in 28 centres in France and enrolled patients both retrospectively and prospectively. Data were analysed from patients who received IVT-AFL treatment between January 1, 2014 and December 31, 2015. The date of the first IVT-AFL injection was considered the baseline visit, and data collection continued for a maximum of 12 months after the first injection. For retrospectively enrolled patients, the number of office visits, eye exams and treatments were collected from medical records; for prospectively enrolled patients, this information was recorded at routine follow-up visits. The study protocol was approved by a French data privacy committee (Comité Consultatif sur le Traitement de l’Information en Matière de Recherche dans le Domaine de la Santé and Commission Nationale de l’Informatique et des Libertés). All patients provided written informed consent to participate.

### Participants

Patients diagnosed with nAMD who had been treated with ranibizumab for > 3 but < 12 months and switched to prescribed IVT-AFL by their physician were included. Eligible patients must have been refractory to ranibizumab, defined as persistent IRF and/or SRF on optical coherence tomography despite treatment with ranibizumab, in accordance with the Haute Autorité de Santé recommendations of at least three injections of ranibizumab.

Patients excluded from the study were those with any ranibizumab-treated eyes for nAMD previously switched to IVT-AFL, absence of treatment criteria for IVT-AFL, eyes previously treated with photodynamic therapy, another retinal disease (diabetic retinopathy, diabetic macular oedema, myopia or angioid streaks) or participation in any interventional study.

The safety analysis set (SAS) included all patients who received ≥1 IVT-AFL treatment in any eye. The per-protocol (PP) population was defined as all patients in the full analysis set (FAS) (i.e., BCVA ≥35 letters at baseline, or delay of ≤380 days between first and last ranibizumab injection, or ≥ 3 ranibizumab injections), with BCVA and central retinal thickness (CRT) assessments prior to any treatment (including ranibizumab), at baseline, and at Month 12. The FAS included patients who received ≥1 IVT-AFL treatment and had BCVA and CRT assessments in the study eye at baseline and during follow-up. Patients from the FAS who received a BCVA/CRT assessment before any treatment (including ranibizumab) at enrolment and at Month 12 were included in the subgroup analysis. This subset of patients was stratified by whether or not they received three initial monthly IVT-AFL injections following the switch.

### Outcomes

The primary endpoint was treatment success rate at 12 months (defined as a gain of ≥1 letter in BCVA and/or any decrease in CRT [in μm] between initial visit [first injection of IVT-AFL] and 12-month follow-up visit). BCVA was measured using ETDRS letters (preferentially) or any other visual scale. For data analysis, we transformed any other visual acuity score to ETDRS letter score.

Secondary outcomes included change in BCVA between baseline and final study visits (12 months after the first injection of IVT-AFL or study discontinuation), mean duration of ranibizumab treatment before initiation of IVT-AFL, and frequency and mean number of IVT-AFL injections over the study period. No BCVA data were collected at the end of ranibizumab treatment; however, switching to IVT-AFL occurred relatively soon after the last ranibizumab injection (median of 44.0 days). Therefore, the change in BCVA during ranibizumab treatment was estimated based on the change between BCVA values before any treatment and before first injection of IVT-AFL.

All adverse events (AEs) occurring after the first IVT-AFL injection were documented in the electronic case report form. All patient medical records were evaluated for demographic as well as clinical characteristics, and AEs were summarised using the Medical Dictionary for Regulatory Activities coding system. The event rates for single AEs were calculated based on the total number of documented patients and AEs were categorised according to connection with medication, seriousness, discontinuation of therapy and outcome.

### Statistical analyses

A sample size of 225 patients was determined based on a 2-sided 95% confidence interval (CI) with a width equal to 0.12 when the sample proportion was 0.70. Primary analysis criteria (success rate) considered patients who discontinued IVT-AFL treatment prematurely to be treatment failures. We expressed the incidence of treatment success at 12 months as number and percentage of patients (n [%]) and provided a 2-sided 95% CI.

The main analysis of secondary criteria (BCVA and CRT) was performed without replacing missing values at study end. Change in BCVA (ETDRS letters) was expressed as mean (standard deviation [SD]) and provided a 2-sided 95% CI. We performed two sensitivity analyses with two imputation methods for missing data: imputation of missing value by the last observation carried forward (LOCF) method and imputation of missing data with the median value of population. Statistical analyses were conducted with SAS software release 9.4 (SAS Institute Inc., Cary, NC, USA).

## Results

### Participants

**Fig. 1:**
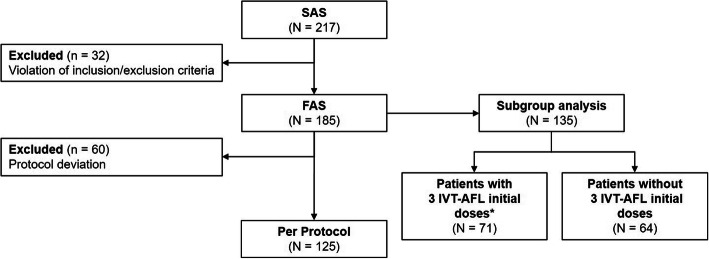
Patient disposition. *Three initial monthly injections (− 1/+ 2 weeks). The per-protocol population was defined as patients without protocol deviation and with BCVA/CRT assessments prior to any treatment (ranibizumab) at baseline and at Month 12 (*n* = 125). The FAS included patients who received ≥1 IVT-AFL treatment and had BCVA and CRT assessments in the study eye at baseline and during follow-up (*n* = 185). BCVA = best-corrected visual acuity; CRT = central retinal thickness; FAS = full analysis set; IVT-AFL = intravitreal aflibercept; SAS = safety analysis set

A total of 236 patients were screened. Of these, 217 were included in the SAS and 125 were included in the PP population (Fig. 1). Demographic characteristics are shown in Table 1.

**Table 1:** Demographic Characteristics

	Full Analysis Set (***n*** = 185)	Per-Protocol Set (***n*** = 125)
**Characteristic at baseline**		
Age, years	77.6 (8.5)	78.2 (8.0)
Female, *n* (%)	109 (58.9)	79 (63.2)
Duration of nAMD, months	8.5 (7.3)	7.4 (3.3)
**Characteristic before any treatment, including ranibizumab**		
BCVA, ETDRS letters	62.4 (16.6)^a^	64.0 (15.6)
CRT, μm	368.3 (124.4)^b^	362.4 (115.2)^c^
Subretinal fluid, *n* (%)	133 (76.9)^b^	92 (74.2)^c^
Intraretinal fluid, *n* (%)	76 (43.9)^b^	55 (44.4)^c^
Pigment epithelium detachment, *n* (%)	125 (72.3)^b^	88 (71.0)^c^

Mean (standard deviation) unless otherwise stated

^a^*n* = 163; ^b^*n* = 161; ^c^*n* = 116; values correspond to assessments before any treatment, including ranibizumab, had been administered

*BCVA* Best-corrected visual acuity, *CRT* Central retinal thickness, *ETDRS* Early Treatment Diabetic Retinopathy Study, *nAMD* Neovascular age-related macular degeneration

### Ranibizumab treatment and outcomes prior to switching

**Fig. 2:**
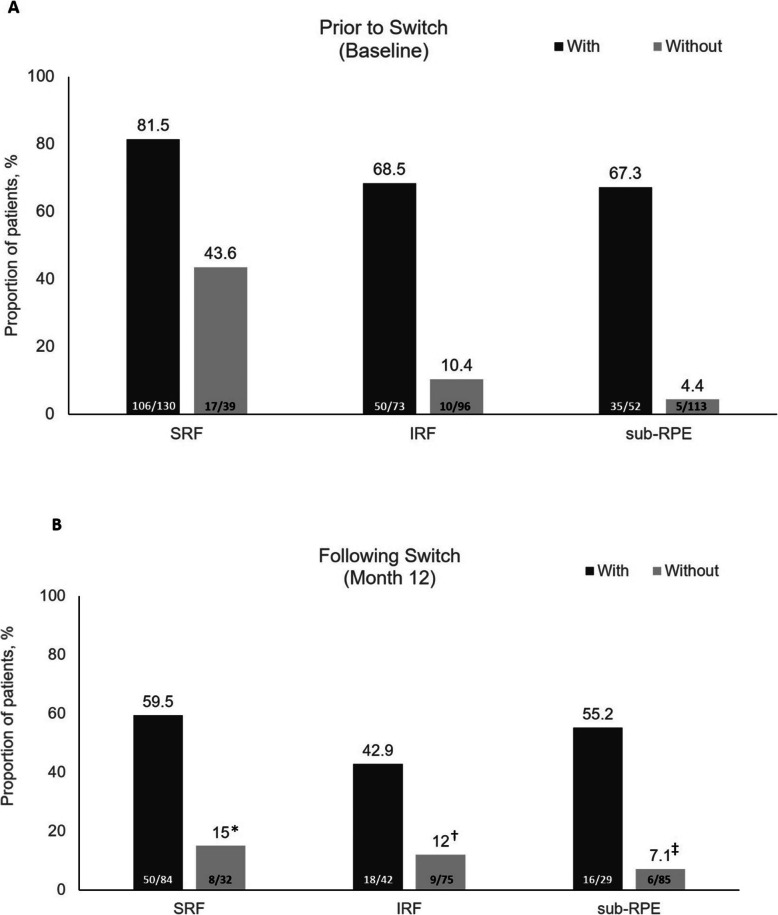
Proportion of patients with SRF, IRF and sub-RPE. At baseline (prior to switch) (**a**) and at Month 12 (following switch) (**b**). IRF = intraretinal fluid; SRF = subretinal fluid; sub-RPE = sub-retinal pigment epithelium. Any treatment includes ranibizumab. *Twelve patients without SRF at baseline were found with SRF at least once over the follow-up and three of them switched from IVT-AFL to ranibizumab. ^†^Twenty-three patients without IRF at baseline were found with IRF at least once over the follow-up and one of them switched from IVT-AFL to ranibizumab. ^‡^Thirteen patients without sub-RPE fluid at baseline were found with sub-RPE fluid at least once over the follow-up and two of them switched from IVT-AFL to ranibizumab

Treatment with ranibizumab was initiated soon after diagnosis of nAMD, with a mean lapse of < 1 month (Table 2). Approximately 75% of patients had their first injection of ranibizumab < 0.3 months after diagnosis of nAMD. Patients received a mean (SD) of 5.1 (2.5) injections over 5.6 (4.3) months [22]. Approximately 50% of patients received ≥4 ranibizumab injections over a median duration of 4.6 months. Mean (SD) BCVA improved significantly with ranibizumab treatment prior to the switch (+ 2.2 [12.0] ETDRS letters, *P* = 0.046) (Table 2). Distribution of patients with SRF, IRF and subretinal pigment epithelium (sub-RPE) at baseline according to the absence or presence of SRF, IRF and sub-RPE before treatment (including ranibizumab), is shown in Fig. 2.

**Table 2:** Ranibizumab Treatment and Outcomes Before Switch to Intravitreal Aflibercept

	Patients (***n*** = 125)
Delay between diagnosis of nAMD and first injection of ranibizumab, months	0.5 (1.2)
Number of ranibizumab injections	4.8 (1.9)
Duration of ranibizumab treatment, months	4.9 (2.8)
BCVA before any treatment (including ranibizumab), ETDRS letters	64.0 (15.6)
BCVA at baseline (switch to IVT-AFL), ETDRS letters	66.2 (12.1)
Change in BCVA from before the start of any treatment (including ranibizumab) and baseline (switch to IVT-AFL)	2.2 (12.0)
* P* value	0.046

All values are expressed as mean (standard deviation); per-protocol population

*P* value is for paired sample *t* test

*BCVA* Best-corrected visual acuity, *ETDRS* Early Treatment Diabetic Retinopathy Study, *IVT-AFL* Intravitreal aflibercept, *nAMD* Neovascular age-related macular degeneration

### Intravitreal aflibercept treatment and outcomes following the switch

The success rate (proportion of patients with a gain of ≥1 letter in BCVA and/or any decrease in CRT [in μm] between baseline [the date of the first IVT-AFL injection] and 12-month follow-up visit) was 68.8%. The mean BCVA improvement from baseline to Month 12 was 1.5 letters (*P* = 0.105) (Table 3). At Month 12, 55.2% of patients (*n* = 69/125) were able to read ≥70 letters.

**Table 3:** Intravitreal Aflibercept Treatment and Outcomes Following Switch to Intravitreal Aflibercept

	Patients (***n*** = 125)
Delay between last injection of ranibizumab and first IVT-AFL, days	61.2 (46.1)
Reasons for switch to IVT-AFL, *n* (%)	
Refractory	122 (97.6%)
AE/SAE	3 (2.4%)
Other	0 (0%)
Time between diagnosis of nAMD and first IVT-AFL, months	7.4 (3.3)
Duration of IVT-AFL treatment, months	11.3 (3.1)
Follow-up duration, months	12.7 (2.0)
Number of IVT-AFL injections	7.5 (2.6)
**BCVA, ETDRS letters**	
Baseline (switch to IVT-AFL)	66.2 (12.1)
Month 12	67.7 (13.6)
Change in BCVA score between baseline and 12 months	1.5 (10.3)
*P* value	0.105
**CRT, μm**	
Baseline (switch to IVT-AFL)	331.2 (103.3)
Month 12	286.2 (84.7)
Change in CRT between baseline and 12 months	−45.0 (101.1)
*P* value	< 0.001
**Patients with SRF,** ***n*** (%)	
Baseline	87 (70.7)
Month 12	58 (48.3)
**Patients with IRF,** ***n*** (%)	
Baseline	44 (35.8)
Month 12	27 (22.5)

All values are reported as mean (standard deviation) unless otherwise indicated; per-protocol population

*P* values are for the paired samples *t* test

*AE* Adverse event, *BCVA* Best-corrected visual acuity, *CRT* Central retinal thickness, *ETDRS* Early Treatment Diabetic Retinopathy Study, *IRF* Intraretinal fluid;IVT-AFL Intravitreal aflibercept, *nAMD* Neovascular age-related macular degeneration, *SAE* Serious adverse event, *SRF* Subretinal fluid

Data on the timing of first injection, treatment duration and number of IVT-AFL injections are shown in Table 3. Given the presence of extreme values, the median data is presented instead of the mean. The delay between the last injection of ranibizumab and the first IVT-AFL injection ranged from 9 to 314 days (mean [SD], 61.2 [46.1]; median 43 days). The main reason for switching from ranibizumab to IVT-AFL was that the patient was considered refractory to ranibizumab.

**Fig. 3:**
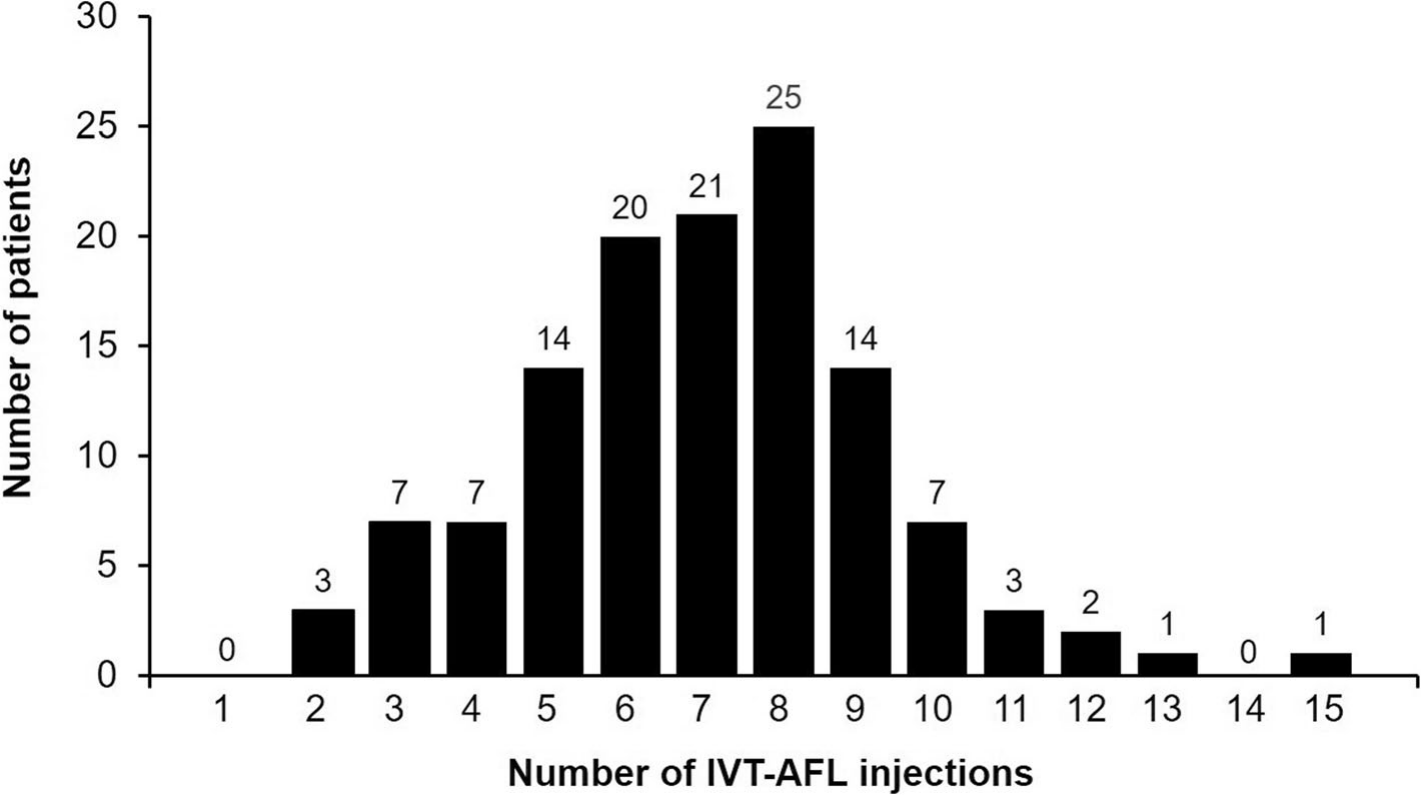
Distribution of number of intravitreal aflibercept injections over the 12-month study. Per-protocol population. IVT-AFL = intravitreal aflibercept

The range of IVT-AFL treatments received over the 12-month study duration is shown in Fig. 3. More than half of patients (52.8%) received three initial monthly IVT-AFL injections. Overall, 17.6% (*n* = 22) of patients switched at least once from IVT-AFL to ranibizumab, and 5.6% (*n* = 7) switched back to IVT-AFL.

### Anatomic outcomes following the switch

Anatomic outcomes improved in patients who were refractory to ranibizumab and switched to IVT-AFL (Table 3). Mean CRT reduction from baseline to Month 12 was 45.0 μm (*P* <  0.001). The proportion of patients with SRF at baseline was 70.7% (*n* = 87/123) and at Month 12 was 48.3% (*n* = 58/120). The proportion of patients with IRF was 35.8% at baseline (*n* = 44/123) and 22.5% (*n* = 27/120) at Month 12, respectively. Differences between the proportions of patients with and without fluid at baseline and Month 12 were significant for SRF and IRF (McNemar test *P* <  0.001 and *P* = 0.014, respectively), but not sub-RPE. Distribution of patients with SRF, IRF and sub-RPE at Month 12 according to the absence or presence of SRF, IRF and sub-RPE at baseline are shown in Fig. 2.

**Fig. 4:**
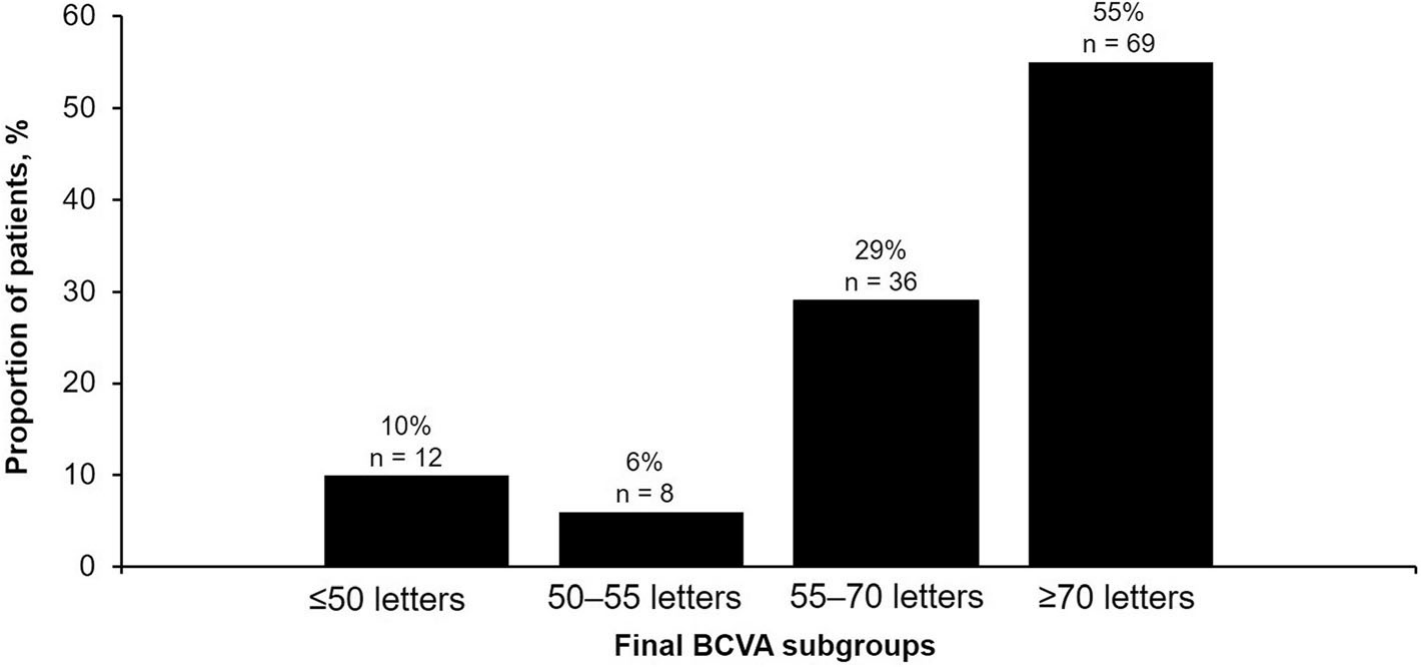
Distribution of patients by absolute final BCVA subgroups (< 50, 50–55, 55–70 and ≥ 70 letters). *n = 125. Per-protocol population. BCVA = best–corrected visual acuity

Approximately one-quarter of patients (24.0%; *n* = 30/125) gained 0 to 4 letters; 16.8% (*n* = 21/125) gained 5 to 9 letters; 10.4% (*n* = 13/125) gained 10 to 14 letters; and 11.2% (*n* = 14/125) gained ≥15 letters from baseline to Month 12. Among patients gaining ≥15 letters, mean BCVA change was 20.6 (8.0) letters. Conversely, 8.0% (*n* = 10/125) lost ≥15 letters. Figure 4 shows the distribution of patients by final absolute BCVA subgroups (< 50; 50 to 55; 55 to 70; and ≥ 70 letters).

### Exploratory and subgroup analyses following switch

In an exploratory analysis conducted in the PP population, patients with < 3 months of ranibizumab treatment prior to baseline gained a mean (SD) of 4.0 (11.1) letters from baseline to Month 12 (*P* = 0.025). This gain was greater than that observed in patients with 3 to 5 months, 6 to 8 months, and ≥ 9 months of ranibizumab treatment prior to baseline (+ 0.1 [10.8], − 0.6 [8.2] and + 2.1 [7.8] letters, respectively) (Supplementary Fig. 1).

In a subgroup analysis in the FAS population (patients with BCVA/CRT assessment before any treatment [including ranibizumab], at baseline and at Month 12), the overall success rate was 66.7%, with higher rates in patients who received three initial monthly IVT-AFL injections compared with those who did not (69.0% vs 64.1%, respectively; Table 4). No statistical test was performed, but 95% CIs largely overlap.

**Table 4:** Success Rate by Use or Non-use of 3 Initial Monthly Injections (FAS Population)

	Overall (***n*** = 135)	With 3 initial monthly injections (***n*** = 71)	Without 3 initial monthly injections (***n*** = 64)
Success rate at 12 months,^a^ *n* (%) [95% CI]	90 (66.7) [58.0–74.5]	49 (69.0) [56.9–79.5]	41 (64.1) [51.1–75.7]
**BCVA, ETDRS letters**			
Baseline	65.1 (13.7)	63.6 (12.3)	66.9 (15.1)
Month 12	66.9 (15.0)	66.8 (12.1)	66.9 (17.8)
Change in BCVA between initial visit and 12 months	1.7 (10.5)	3.3 (11.0)	0.0 (9.6)
*P* value	0.056	0.015	NS
**CRT, μm**			
Baseline	328.0 (100.9)	338.6 (114.6)	316.3 (82.5)
Month 12	285.3 (84.3)	296.1 (88.0)	272.6 (78.6)
Change in CRT between initial visit and 12 months	−47.0 (104.1)	−45.5 (106.0)	−48.8 (102.6)
*P* value	< 0.001	< 0.001	< 0.001

All values are reported as mean (standard deviation) unless otherwise indicated

*P* values are for the paired samples *t* test

^a^Patients who gained ≥1 ETDRS letter (BCVA) and/or a reduction in CRT from baseline (prior to switch) to 12 months after the first intravitreal aflibercept injection

*BCVA* Best–corrected visual acuity, *CI* Confidence interval, *CRT* Central retinal thickness, *ETDRS* Early Treatment Diabetic Retinopathy Study, *FAS* Full analysis set, *NS* Not significant

At Month 12, in patients who received three initial monthly IVT-AFL injections, mean BCVA gain was 3.3 letters (*P* = 0.015). Mean BCVA was stable in patients who did not receive three initial monthly injections (Table 4). There was a trend in favour of higher BCVA gains among patients with lower initial BCVA.

### Sensitivity analyses

Results of the sensitivity analyses were consistent with the results of the main analyses. Specifically, the success rate in the overall population was 68.8% when we replaced missing data by the median of the population, or by LOCF. Mean (SD) gain in BCVA was 1.5 (10.3) letters (*P* = 0.105) when missing data were replaced by the median of the population, or by LOCF. At Month 12, the proportion of patients who could read ≥70 letters was 55.2% for median value replacement and for LOCF value replacement.

### Safety outcomes

Excluding lack of efficacy and inappropriate scheduling of drug administration, which were also considered AEs according to the protocol and occurred in 12.4 and 5.1% of patients, respectively, the most common AE was eye pain (2.3%) (**Table 5**). Treatment-emergent serious AEs were reported in 1.8% of patients, none associated with IVT-AFL treatment. The most common non-ocular AE was falls, occurring in 1.4% of patients. Twenty-seven patients discontinued IVT-AFL due to lack of efficacy and a further seven discontinued IVT-AFL due to other treatment-emergent AEs; in two patients, the AEs (both PED, one with retinal exudate) were considered related to IVT-AFL.

## Discussion

Results from the TITAN study indicate that approximately two-thirds (67.7%) of patients with nAMD who switched from ranibizumab to IVT-AFL treatment (after ≤12 months of treatment with ranibizumab) achieved success, defined as a gain of ≥1 letter in BCVA and/or any decrease in CRT between baseline and Month 12. Patients considered refractory to first-line treatment with ranibizumab had an additional gain (beyond the mean 2.2 letters initially achieved with ranibizumab) of 1.5 letters at Month 12 after IVT-AFL treatment was initiated, indicating that switching from ranibizumab to IVT-AFL can be considered in ranibizumab-refractory nAMD. There was an inverse relationship between baseline BCVA and visual acuity gain at Month 12. Generally, patients with lower baseline BCVA had greater visual acuity gains at Month 12. Patients who had previously received ranibizumab for < 3 months prior to switch had a greater improvement in BCVA. In addition to decreases in CRT after switching treatment, an enhanced anatomic response was observed, with reductions in fluid accumulation observed in the SRF, IRF and sub-RPE compartments. Overall, these results suggest that considering a switch to IVT-AFL from ranibizumab after < 3 months may be warranted; however, these findings should be interpreted with caution as the TITAN study was not specifically designed to answer this question, and other confounding variables such as duration of disease are likely to have had an effect.

Findings from other studies of patients refractory to ranibizumab, who switched to IVT-AFL after varying durations of treatment, suggest that there may be a benefit to switching, particularly with anatomical benefit after the switch in terms of improvements in central retinal thickness and pigment epithelium detachment [23]. In fact, the effect of switching on functional outcomes has been shown to be variable [23] and, in our analysis, the overall mean change in BCVA was relatively small. However, over 20% of the ranibizumab-refractory patients achieved BCVA gains of ≥10 letters after receiving IVT-AFL, suggesting there may be specific subgroups of patients who could respond particularly well to switching to IVT-AFL, despite an initially poor response to anti-VEGF therapy with ranibizumab. Further randomised, controlled studies with appropriate control groups are, however, required.

The present study highlights the importance of three initial monthly injections at the start of IVT-AFL treatment. Greater visual improvements were observed in patients who received three initial monthly injections than in those who did not, which is consistent with previous studies of patients with nAMD who were switched from ranibizumab to IVT-AFL [21, 24]. It is notable that the proportion of patients who received three initial monthly injections in TITAN was lower (52.8%) than expected given the recommended dosing regimen in France [25]. Findings from this study were consistent with the known safety profile of IVT-AFL in nAMD [13, 26].

A few limitations inherent in the observational study design should be noted, including the use of different charts to evaluate visual acuity. Here, ETDRS letter charts or any other visual scale were used to evaluate visual acuity; if the latter, results were converted to ETDRS letters, potentially introducing a bias, especially when measuring the number of letters gained or lost after treatment. Also, our study design includes both retrospective and prospective components, and lacks a control group, which is a fundamental aspect of an observational study. In addition, findings are from a single European country, which may not be representative of other countries. Some data were imputed due to variability in data collection; a feature common in observational studies. Given that success was not achieved if patients discontinued IVT-AFL prematurely, even if they switched back to IVT-AFL at a later point, the proportion of patients achieving success may have been underestimated. The success rate was 70.3% (FAS) using median data. Patients who switched from IVT-AFL prior to receiving 12 months of treatment were considered failures; therefore, replacement of missing data by median value of population only slightly affected the success rate. Finally, factors that could influence differences in visual and anatomic outcomes between groups (e.g., disease severity at baseline, frequency of injections and physician monitoring) were not explored in the TITAN study.

## Conclusions

The TITAN study demonstrated the effectiveness and safety of IVT-AFL in patients with ranibizumab-refractory nAMD in routine clinical practice in France. These findings suggest that an early switch (< 12 months) from ranibizumab to IVT-AFL may improve visual outcomes over 12 months for some patients in this difficult-to-treat population. The study further highlighted that initiating IVT-AFL treatment with three initial monthly injections after switching from ranibizumab may improve visual outcomes. Further studies with appropriate control groups are required to understand how to best identify those patients most likely to benefit from switching to IVT-AFL.

## Supplementary Information

**Additional file 1: Supplementary Figure 1**. Visual gains in patients treated with ranibizumab prior to switching to IVT-AFL. Per-protocol population. BCVA = best–corrected visual acuity; IVT-AFL, intravitreal aflibercept.

## Data Availability

Availability of the data underlying this publication will be determined according to Bayer’s commitment to the EFPIA/PhRMA “Principles for responsible clinical trial data sharing”. This pertains to scope, time point and process of data access. As such, Bayer commits to sharing, upon request from qualified scientific and medical researchers, patient-level clinical trial data, study-level clinical trial data, and protocols from clinical trials in patients for medicines and indications approved in the United States (US) and European Union (EU) as necessary for conducting legitimate research. This applies to data on new medicines and indications that have been approved by the EU and US regulatory agencies on or after January 1, 2014. Interested researchers can use www.clinicalstudydatarequest.com to request access to anonymised patient-level data and supporting documents from clinical studies to conduct further research that can help advance medical science or improve patient care. Information on the Bayer criteria for listing studies and other relevant information is provided in the Study sponsors section of the portal.

## Abbreviations

AE: Adverse event
anti-VEGF: Anti-vascular endothelial growth factor
BCVA: Best-corrected visual acuity
CRT: Central retinal thickness
ETDRS: Early Treatment Diabetic Retinopathy
FAS: Full analysis set
IRF: Intraretinal fluid
IVT-AFL: Intravitreal aflibercept
LOCF: Last observation carried forward
nAMD: Neovascular age-related macular degeneration
PP: Per-protocol
SAS: Safety analysis set
SD: Standard deviation
SRF: Subretinal fluid
sub-RPE: Subretinal pigment epithelium
