# Supplementary figures and images for: Efficacy and safety of intravitreal aflibercept in ranibizumab-refractory patients with neovascular age-related macular degeneration
Source: BMC Ophthalmol. 2021 Feb 17;21:90. doi: 10.1186/s12886-021-01841-6 (PMC7890834; doi:10.1186/s12886-021-01841-6)

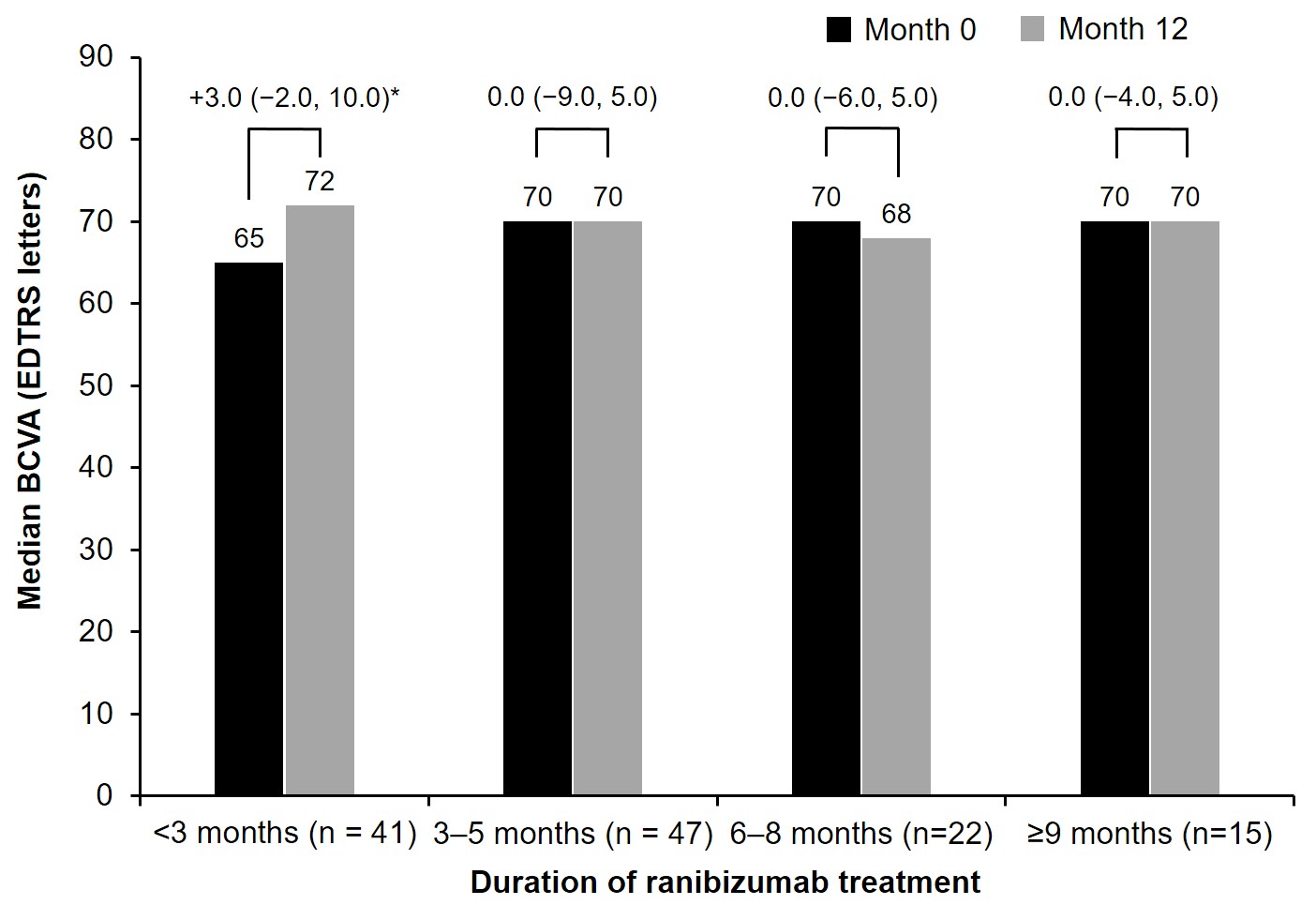

Supplement: Supplementary file 1 — Additional file 1: Supplementary Figure 1. Visual gains in patients treated with ranibizumab prior to switching to IVT-AFL. Per-protocol population. BCVA = best–corrected visual acuity; IVT-AFL, intravitreal aflibercept. [file 12886_2021_1841_MOESM1_ESM.jpg]
